# Response to intruder number is related to spontaneous quantity discrimination performance in a wild bird

**DOI:** 10.1093/beheco/araf093

**Published:** 2025-08-20

**Authors:** Grace Blackburn, Benjamin J Ashton, Holly Hunter, Amanda R Ridley

**Affiliations:** Centre of Evolutionary Biology, School of Biological Sciences, University of Western Australia, 35 Stirling Highway, Crawley 6009, Australia; Coastal Marine Ecosystems Research Centre, Central Queensland University, 554/700 Yaamba Road, Norman Gardens, QLD 4701, Australia; College of Science and Engineering, Flinders University, Sturt Road, Bedford Park, SA 5042, Australia; Centre of Evolutionary Biology, School of Biological Sciences, University of Western Australia, 35 Stirling Highway, Crawley 6009, Australia; Centre of Evolutionary Biology, School of Biological Sciences, University of Western Australia, 35 Stirling Highway, Crawley 6009, Australia

**Keywords:** bird, cognition, intergroup interaction, playback, quantity discrimination, wildlife

## Abstract

Quantity discrimination abilities are considered a valuable skill for many aspects of life, including foraging, predator avoidance, and intergroup contests. Two types of experiments are often utilized to detect such abilities in animals; cognitive tasks in which individuals must choose between two quantities of food, and playback experiments of the vocalizations of differing numbers of intruding individuals. To date, no study has investigated whether individual performance in these two types of experiments is related. We presented wild Western Australian magpies (*Gymnorhina tibicen dorsalis*) with both a spontaneous quantity discrimination cognitive task and a playback experiment, to investigate quantity discrimination abilities, and to explore if performance on these experiments is related. We found that magpies (1) selected the greater quantity of food in the cognitive task and (2) responded more strongly to playback of three callers compared to one caller, suggesting this species possesses quantity discrimination abilities. Individual performance on these two experiments was negatively correlated, with magpies that performed better on the cognitive task spending less time vigilant following the three-caller playback compared to magpies that performed worse. Our results highlight the importance of exploring the relationship between performance in a cognitive task and ecologically relevant behaviors, as this has the potential to offer profound insights into cognitive ecology.

## Teaser

The ability to discriminate between quantities is important for animal species. We show that wild magpies can discriminate both between different quantities of a food item, choosing the larger amount of food more often than at chance, and different numbers of callers, showing more vigilance following the song of more calling magpies. Notably, we also found that performance on these two experiments was related, with birds that performed better on the food task spending less time vigilant following playback of three callers, highlighting the importance of quantity discrimination abilities for magpies.

## Introduction

The ability to assess and discriminate between quantitative differences is a cognitive skill considered important for many aspects of animal life (Benson-Amram et al. 2017; Nieder 2020). In line with optimal foraging theory (Krebs et al. 1974; Nieder 2020), when faced with a choice between foraging patches that differ in the quantity of food, individuals will likely benefit from selecting a foraging patch with more food, as has been observed in many species (Uller et al. 2003; Bogale et al. 2014; Szabo et al. 2021). For prey species, such as angelfish (*Pterophyllum scalare*) and three-spined sticklebacks (*Gasterosteus aculeatus*), the ability to assess quantitative differences and select the larger of two groups when choosing a shoal to join likely reduces predation risk (Gómez-Laplaza and Gerlai 2011; Mehlis et al. 2015). Similarly, in both great tits (*Parus major*) and jackdaws (*Corvus monedula*) the ability to assess the number of callers is an important determinant of anti-predator mobbing behavior (Coomes et al. 2019; Dutour et al. 2021). In these species, the likelihood of an individual joining a mobbing event is greater if more individuals are already producing mobbing calls, as more individuals mobbing equates to less risk per individual (Coomes et al. 2019; Dutour et al. 2021). Similar abilities may also be beneficial for the hosts of brood parasites. For example, American coots (*Fulica americana*) are able to count their eggs and subsequently detect brood parasitism (Lyon 2003). Accordingly, quantity discrimination abilities are widespread and have been observed in mammals (McComb et al. 1994; Uller and Lewis 2009; Benson-Amram et al. 2011, 2017), birds (Radford 2003; Garland et al. 2012; Bogale et al. 2014), reptiles (Miletto Petrazzini et al. 2017; Szabo et al. 2021, 2023; Tomonaga et al. 2023), amphibians (Uller et al. 2003; Stancher et al. 2015; Khatiwada and Burmeister 2022), fish (Agrillo et al. 2008; Gómez-Laplaza and Gerlai 2011), and insects (Howard et al. 2020; Bengochea et al. 2023).

One important application of quantity discrimination abilities for social species is during intergroup contests (McComb et al. 1994; Radford 2003; Seddon and Tobias 2003; Benson-Amram et al. 2011, 2017). When intergroup contests are costly, competitors should assess the resource holding potential (RHP) of their opponents and evaluate the likelihood of winning (or losing) the fight prior to investing in it (Parker 1974; Maynard Smith 1982). In group-living species, differences in group size may be a more accurate determinant of the outcome of a contest than individual attributes (McComb et al. 1994; Radford 2003; Seddon and Tobias 2003). Accordingly, several studies on territorial group-living species (both observational studies of natural intergroup contests, and playback experiments using simulated intruders) have shown the number of intruding individuals to affect individual or group response to these intruders (McComb et al. 1994; Seddon and Tobias 2003; Benson-Amram et al. 2011; Van Belle and Scarry 2015). For example, in the subdesert mesite (*Monias benshi*), the strength of response to playback of intruding groups was significantly affected by the number of simulated intruders, with groups less likely to approach the speaker as the number of simulated intruders increased (Seddon and Tobias 2003). Similar findings have been reported in lions (*Panthera leo*) (McComb et al. 1994), spotted hyenas (*Crocuta crocuta*) (Benson-Amram et al. 2011), black howler monkeys (*Alouatta pigra*), and tufted capuchin monkeys (*Sapajus nigritus*) (Van Belle and Scarry 2015), providing evidence for quantity discrimination abilities in these species.

There are two cognitive systems thought to be used by animals in the processing of numerical stimuli (Hyde 2011; Agrillo 2015). The first is known as the object-file or object-tracking system (OFS) (Hyde 2011; Agrillo 2015). In the OFS, animals individuate each object, allocating a distinct mental symbol to each as it is introduced (Rugani 2017). The OFS is typically limited to between one and four items, as it is defined by the number of symbols that can be held concurrently in the working memory (Hyde 2011; Rugani 2017). The second system is the approximate number system (ANS) and is an inexact system that is thought to be used when processing larger quantities (Hyde 2011; Khatiwada and Burmeister 2022). Under the ANS, the discrimination of quantities follows Weber's Law, whereby the accuracy of numerical discrimination increases as the ratio decreases (Hyde 2011; Nieder 2020). However, it is important to note that whether these two systems are mutually exclusive or instead operate on a continuum is a source of debate (Cordes and Brannon 2009; Hyde 2011; Stancher et al. 2013; Agrillo and Bisazza 2018).

Research into the numerical abilities of animals generally utilizes either spontaneous tests or training procedures (Agrillo and Bisazza 2014; Agrillo et al. 2017). While both methodological approaches have their issues and advantages, spontaneous choice tests are thought to be more ecologically relevant and focused on the cognitive mechanisms applied under natural situations (Agrillo and Bisazza 2014), and hence these are the focus of the present study. Spontaneous quantity discrimination tasks typically fall into two categories: (i) spontaneous quantity discrimination (hereafter, SQD) tasks, where individuals are presented with two different quantities of food and their choices are assessed (Uller et al. 2003; Uller and Lewis 2009; Bogale et al. 2014; Miletto Petrazzini et al. 2017; Howard et al. 2020; Szabo et al. 2021, 2023; Khatiwada and Burmeister 2022; Tomonaga et al. 2023); or (ii) playback experiments, where variable numbers of callers are broadcast to individuals or groups and their subsequent responses are measured (McComb et al. 1994; Radford 2003; Seddon and Tobias 2003; Bradley and Mennill 2009; Benson-Amram et al. 2011; Benson-Amram et al. 2017; Coomes et al. 2019; Scarry 2020; Dutour et al. 2021). SQD tasks usually focus on foraging decisions and present an artificial binary choice between two quantities of food, while playback presentations often focus on contests between conspecific groups and present individuals with ecologically relevant scenarios reflective of real intergroup interactions. It is often assumed that the same quantity discrimination abilities underlie the decisions of individuals in both types of experiments (Benson-Amram et al. 2017; Nieder 2020), yet, to our knowledge, no study has investigated if there is a link between performance in these two types of experiments. Investigating the relationship between performance on cognitive tasks and behavior in ecologically relevant situations (as can be simulated by playback presentations) has the potential to reveal important insights into cognitive ecology and can help to explore the ecological relevance of such tasks as well as the cognitive traits underpinning them.

Here, we presented SQD tasks and playback experiments to wild group-living Western Australian magpies to investigate (i) whether this species is capable of discriminating between different quantities of food items, (ii) whether this species is able to discriminate between different numbers of callers, and (iii) whether performance in these two experiments is related. We predict that (i) magpies will be able to discriminate between different quantities of food items, (ii) magpies will be able to discriminate between playbacks of different quantities of callers, and (iii) performance in a quantity discrimination food task will be related to how individuals respond to playbacks of different quantities of callers. Specifically, we predict that individuals that perform better on the SQD task (ie are better able to discriminate between differing amounts of food) will respond more strongly to playback of multiple callers compared to individuals that perform worse on the SQD task, as these better performing individuals are more able to discern that these tracks represent multiple intruding individuals.

## Methods

### Study species

Western Australian magpies (*Gymnorhina tibicen dorsalis*) are a cooperative breeding, group-living passerine found in the southwest of Western Australia (Johnstone 2004; Pike et al. 2019). Western Australian magpies are sexually dichromatic, allowing sex to be visually discerned once individuals have reached their full adult plumage, at approximately 3 years of age (Edwards et al. 2015). The Western Australian subspecies lives in highly territorial groups that defend their territories year-round via vocal and visual displays, and often engage in high risk contests with other groups over territory, mates, and other resources (Hughes et al. 2003; Hidayat 2018). During territorial disputes, magpies produce carols, a high amplitude territorial song (Farabaugh et al. 1992; Dutour and Ridley 2020). Carols of multiple individuals together create choruses (Carrick 1963; Dutour and Ridley 2020), a central element of intergroup contests in this species. Magpies often forage far apart from group members, and therefore individuals within a group may experience different rates of intergroup interactions (personal observation G.B. & A.R.R. 2021–2024).

This study was conducted between March and July in 2022 and 2023 on an established study population of Western Australian magpies located in Crawley and Guildford, Perth. Individuals within this population are habituated to human presence, and many birds in this population have undergone testing on cognitive tasks previously (Ashton et al. 2018; Blackburn et al. 2023; Sollis et al. 2023; Hunter et al. 2025). Most magpies in this study population are color ringed, allowing for individual identification, and unringed birds are identifiable via plumage anomalies. Twenty-one birds from nine magpie groups in this population were observed for this study. All fieldwork was conducted between 5am and 11am, when this species is most active (Edwards et al. 2015).

### Spontaneous quantity discrimination (SQD) task

**Fig. 1.**
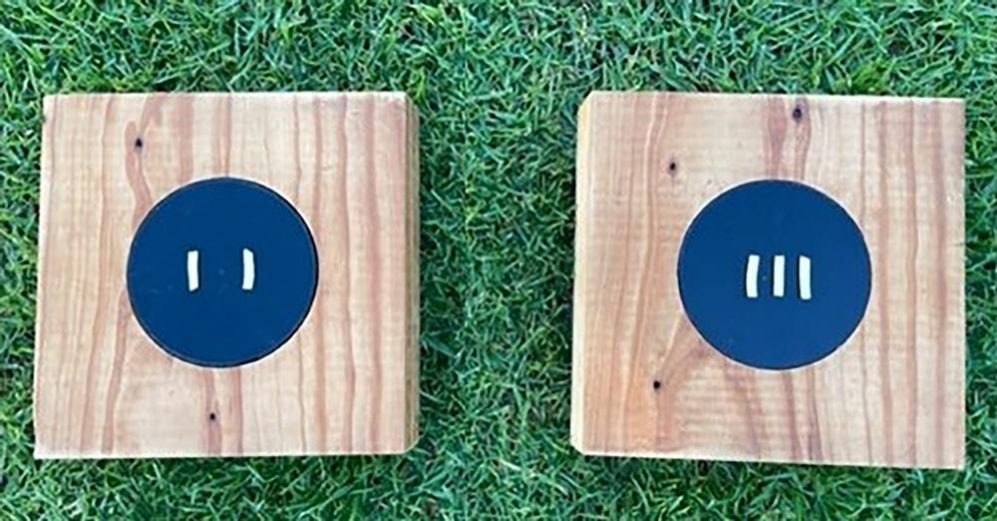
SQD task presenting the 2 vs 3 ratio. Lids were marked identically with six small lines, 1 cm apart. To control for horizontal space and ensure birds were not selecting the food reward that represented the greatest amount of horizontal space, the two strands of cheese (on the left board) were placed between the 1st and 2nd, and 5th and 6th marked lines, and the three strands of cheese (on the right board) were placed between the first and second, third and fourth, and fifth and sixth marked lines.

The SQD task comprised two identical wooden boards (20 cm L × 20 cm W × 4.5 cm H) each containing a central well (10 cm diameter, 3 cm deep). This well was covered with a black lid that could be removed by experimenters but was immovable to magpies (Fig. 1). Each well was filled with mozzarella cheese before being covered with the black lid, to ensure each board presented similar olfactory cues and therefore to help control for the potential use of these cues when selecting a board. Strands of mozzarella cheese (previously used as a food reward for cognitive testing in this species; Ashton et al. 2018; Blackburn et al. 2022) were cut to 2.5 cm in length, and placed on the two black lids of each board in four different ratios; 2 vs 2 (1), 2 vs 3 (0.66), 2 vs 4 (0.5), and 2 vs 5 (0.4). The black lids were marked identically with six small lines, in the center of the lid and 1 cm equidistant from each other, to mark where cheese strands would be placed. This was done to ensure that the horizontal space that the food reward was presented across remained consistent, and therefore to stop birds from simply selecting the board with the food that took up the most amount of horizontal space.

Each of the four ratios (2 vs 2, 2 vs 3, 2 vs 4, and 2 vs 5) were presented to focal individuals once a day for 15 days, resulting in a total of 60 trials per bird. The order in which the four ratios were presented was randomized each day, as was the side holding the greater number of cheese strands. In each trial, cheese strands were placed on the black lids of the boards, out of view of the focal individual, before placing the boards equidistantly in front of the focal individual, 1 m from the bird, and 30 cm from each other. The experimenter would then step back to approximately 1 to 2 m from the boards, allowing the focal individual to approach the boards and select the food reward from one board (indicated by the bird touching the board and/or consuming a strand of cheese), which was considered the focal individual's choice. Latency to interact with the task was quantified (as a proxy of neophobia) by measuring the time from when the task was placed in front of the bird (1 m from the bird), to when the bird first touched the task. Birds were allowed to consume the entire food reward presented on the board of their choice before both boards were removed. An individual's SQD score was quantified as the number of times they selected the larger amount of food in the 45 trials (excluding the 15 2 vs 2 control trials which were used to assess for the presence of a side bias). A higher SQD score was therefore indicative of better cognitive performance.

Animals may vary in performance on cognitive tasks due to motivation (Boogert et al. 2018), therefore in our 2023 testing period, we also quantified a number of proxies of motivation. We conducted 10 min focal observations of focal birds within the 15 days of cognitive testing for that bird, from which we were able to calculate foraging effort (the time that individual spent foraging over the total focal time), and foraging efficiency (the grams of prey caught by an individual in the time spent foraging, calculated following (Edwards et al. 2015)). Focal observations were conducted prior to cognitive testing to gain a representative measure of motivation during testing, and multiple focals were conducted for most magpies, across which foraging effort and foraging efficiency were averaged (average ± SE = 1.86 ± 0.13 focals per bird). As magpies in our study population have been previously trained to jump onto a top-pan scale for a food reward (Blackburn et al. 2023), we were also able to take body mass measurements for individuals on several days of testing within the 15 d period of cognitive testing for that bird. Body mass, foraging effort, and foraging efficiency measurements for each individual were averaged over the 15 days of testing.

### Quantity discrimination playbacks

#### Call collection and playback preparation

We collected naturally emitted carols and choruses of known individuals and groups of individuals respectively, using a RODE NTG-2 directional microphone set within a blimp suspension windshield system and attached to a Roland R-07 wave/MP3 recorder, set at a sampling rate of 44.1 kHz. Carols and choruses were recorded by an experimenter at a distance of 1 to 5 m from the calling bird/s. For each carol and chorus collected, we noted the individuals that sung in that vocalization, as well as their sex, age (fledgling, juvenile, or adult), and territorial group. Only carols and choruses of adult magpies were used in playback tracks.

Playbacks were prepared using Audacity version 3.0.2. Only carols and choruses that were high-quality (had a high signal to background noise ratio), and during which no other birds or magpies that were not involved in the chorus could be heard, were used for playback preparation. We prepared three types of playbacks: (1) a carol of a single caller, (2) a chorus of two callers, and (3) a chorus of three callers. Each track consisted of five seconds of silence, followed by two seconds of background noise that faded in (during which no birds were heard calling), followed by the carol or chorus that had been normalized to ensure all calls were broadcast at their natural amplitude. Other than this normalization, songs were not altered in any way, and thus reflected natural carols and choruses that magpies would hear in the wild. We measured the amplitude of a subset of carols and choruses using a DIGITECH Micro Sound Pressure Level Meter (hereafter, SPL meter) to ensure that playback tracks were broadcast at a natural amplitude. The mean amplitude (and amplitude range) of one-caller carols, two-caller choruses, and three-caller choruses at 1 m from the calling bird/s was 81.3 dB (range: 74.0 to 86.0 dB, *N* = 6), 81.5 dB (range: 71.9 to 88.2 dB, *N* = 7), and 85.9 dB (range: 81.1 to 91.4 dB, *N* = 8) respectively. An ANOVA comparing amplitude by song type (one-caller carol, two-caller chorus, and three-caller chorus) found no significant difference between the amplitude of each song type (*F* (2,18) = 1.58, *P* = 0.23). Background noise was collected using the same set-up used for carol and chorus collection, and from the territory of each calling group when no loud anthropogenic noise or other bird calls were present. After files were created, they were loaded onto a Fiio M6 portable high resolution music player, which was attached by an aux cord to a UE boom 2 speaker for playback presentations. We then used a SPL Meter to check the amplitude of tracks was the same as the amplitude of naturally occurring magpie carols and choruses at 1 m from the speaker/bird.

#### Playback presentations

Each focal bird was presented with each of the three playback tracks (one-caller, two-callers, and three-callers), presented in a randomized order at least five days apart, to minimize any potential habituation to playback tracks, as well as to minimize stress of birds that may arise due to these simulated intergroup conflicts. Playbacks were always from birds and groups that were unfamiliar to the focal bird (located ≥ 15 km from the territory of the focal bird's group) and were presented in the center of the focal birds' territory. Each playback track presented to the same focal bird consisted of different individuals, to minimize habituation from hearing the same individual in multiple playback tracks. Focal birds were always presented with carols (one-caller track) from the same sex, and with choruses (two- and three-callers) with at least half of the calling birds being the same sex (ie, females were presented with female carols, and choruses with at least one female for the two-caller track, and at least two females for the three-caller track). This was done as it was not always possible to get choruses of two or three callers with all males or females. Sex ratio (the number of calling individuals of the same sex as the focal bird divided by the total number of callers in each playback track) was included as a predictor in playback analysis.

Playbacks only began when the focal bird was in social isolation (with other magpies >10 m away), was foraging in an open area, and was not displaying prolonged vigilance (sensu Blackburn et al. 2023). The UE boom 2 speaker was placed 10 m from the focal bird, and playback was played following ≥1 min during which no other magpie vocalizations or loud noises occurred. The response of the focal bird to playback was video-recorded using a Panasonic HC-V180 video recorder. Video recording began one minute prior to the start of playback and was terminated 2 min post-playback.

Videos were analyzed with the analyst (GB) blind to the playback treatment of each video. From each video, we assessed the following behaviors: the time magpies spent vigilant in the minute preplayback, the time magpies spent vigilant in the 2 min post-playback (2 min post-playback was used as most magpies were still vigilant and/or calling following 1 min post-playback), the number of songs the focal bird produced in the 2 min post-playback, and whether birds approached the speaker in the 2 min post-playback. Birds were considered vigilant when displaying an erect posture and scanning the environment (Zhou et al. 2019; Blackburn et al. 2022).

### Statistical analysis

#### Repeatability of SQD performance

Since many birds in this study population had been previously tested on the same SQD task (Hunter et al. 2025), we were able to quantify repeatability in performance over 2 years of testing (2022 and 2023). Eighteen birds completed testing on this task in both 2022 and 2023, totaling 36 test scores. We compared the total number of times that birds selected the larger food reward (out of 45 trials; 15 trials each on the 2 vs 3, 2 vs 4, and 2 vs 5 ratios) in 2022 and 2023. Repeatability estimates were calculated using the RptR package (Nakagawa and Schielzeth 2010) in R. We fitted generalized linear mixed models (GLMMs) with a Gaussian distribution, SQD score (number of times the larger amount of food is chosen) as the dependent variable, and bird ID as a random term. Group ID was not included as a random term in repeatability models as this resulted in overfitting. All model assumptions were then checked using the DHARMa package (Hartig 2021) in R.

#### SQD task analysis

From the 21 individuals who completed SQD testing in 2023, we first conducted a binomial test using performance on all 2 vs 2 trials to determine whether a side bias existed in the data. 2 vs 2 control trials were subsequently excluded from analysis as we found no side bias (binomial test on 2 vs 2 ratio; *N* = 21, *P* = 0.07), and because these trials represented no difference in quantities. We then conducted binomial tests across each test ratio (2 vs 3 (0.66̅), 2 vs 4 (0.5), and 2 vs 5 (0.4)) to determine if the number of times magpies chose the larger food reward was significantly different from random.

We fitted GLMMs with a binomial distribution (logit link function) to investigate the factors affecting performance in the SQD task. The response term for these models was whether birds chose the larger (1) or smaller (0) quantity of food in each trial. Bird identity and group identity were included as random terms. Predictors for these models included latency to interact with the task, weather (clear/cloudy), temperature, adult group size, sex, body mass, foraging effort, foraging efficiency, and ratio presented (2 vs 3 (0.66̅), 2 vs 4 (0.5), 2 vs 5 (0.4)). Temperature measurements were obtained from the Bureau of Meteorology records from the nearest weather station, ∼4 km from the Guildford field site, and ∼5 km from the Crawley field site (Australian Government Bureau of Meteorology 2023).

#### Playback analysis

To ensure that the time spent vigilant in the minute prior to the beginning of playback was not affected by track order, we fitted GLMMs where the total time recorded prior to playback (60 s) was the binomial denominator, and time individuals spent vigilant in the minute prior to playback (seconds) was the response variable. We included track order, sex, and adult group size as predictor terms. We used GLMMs to investigate the factors affecting the time individuals spent vigilant in the 2 min post-playback. In these models, the total time video recorded following playback (120 s) was the binomial denominator, and time individuals spent vigilant during this time (seconds) was the response variable. To investigate the number of songs sung in the 2 min post-playback, we fitted Poisson GLMMs with a log link function and the number of songs as the response variable. For analysis of the factors affecting whether birds approached the speaker following playback, we fitted a binomial GLMM with a 0, 1 response variable (where 0 = did not approach the speaker, and 1 = did approach the speaker).

Predictor terms for these models included track order, sex, sex ratio (the number of calling individuals of the same sex as the focal bird divided by the total number of callers in each playback track), adult group size of the focal bird, carol/chorus playback length, and playback treatment. All models included bird and group identity as random factors. We then repeated this analysis on a subset of 15 individuals (*N*  *=* 45 playbacks) that had undergone cognitive testing in the SQD in 2023 (the same year as the playback experiment). In this analysis, we added SQD score as a predictor and included a model with the interaction between SQD score and playback treatment. The purpose of this was to investigate if performance on the cognitive task was related to response to playbacks (in the model with SQD score as the sole predictor), and whether this differed between playback treatment (in the model with the interaction between SQD score and playback treatment).

#### Model selection

We used Akaike's Information Criterion values corrected for small sample sizes (AICc) to determine which predictors best explained variation in the data. Models included single, additive, or interacting terms. Models were chosen based on their plausibility as an a priori hypothesis, based on the inference approach presented by Tredennick et al. (2021). Prior to being included in a model, predictors were tested for collinearity using the variance inflation factor (VIF). All VIFs were <2 (a VIF of 2 is considered a high level of correlation; Fox and Monette 1992), therefore all predictors were retained. The AICc of each model was compared to a null model which contained only the intercept and random terms, and a top model set was generated of all models within 2AICc of the top model. If multiple models were within 2AICc of the top model, the simplest model was chosen (Harrison et al. 2018). Terms in these models were considered significant if their parameter 95% confidence intervals (CI) did not intersect zero (Grueber et al. 2011; Symonds and Moussalli 2011). If a predictor when tested alone in a model was not significant, it was not considered for additive models but was considered in interactions if these represented a priori hypotheses.

#### Post hoc comparisons

We used the functions *emtrends* and *emmeans* (within the R package *emmeans* (Lenth 2019)) to obtain contrasts between different levels of playback treatment, and levels of the interaction between playback treatments and SQD scores, in cases where there was a significant interaction between these. *P*-values were adjusted for multiple comparisons via the Tukey method. The *emmeans* function was also used to determine effect sizes for contrasts. For binomial GLMMs, odds ratios (OR) were used, while for Poisson GLMMs, rate ratios were used (Nakagawa and Cuthill 2007; Ivarsson et al. 2013). Effect sizes were considered small if OR was >1.50 (or <0.67), medium if OR was >3.50 (or <0.29), and large if OR was >5.10 (<0.20) (Cohen 1988; Ivarsson et al. 2013).

## Results

### Repeatability of SQD performance

For the 18 birds that had undergone testing in both 2022 and 2023, the average number of times the larger amount of food was chosen was 31.3 out of 45 trials (SE = 1.04) in 2022, and 29.1 out of 45 (SE = 1.13) in 2023. The repeatability analysis revealed that performance on the SQD task was significantly repeatable (*R* = 0.461, CI = 0.06–0.74, *P* = 0.025, *N* = 18 birds) over the 2 years of testing.

### SQD performance

Twenty-one adult magpies were tested on the SQD task in 2023, resulting in a total of 1260 trials (15 trials of each combination per bird).

Magpies chose the larger quantity of food more than would be expected if choosing randomly in the 2 vs 3 (0.66̅) ratio (mean ± SE = 8.67 ± 0.52; binomial test: *N* = 21, *P* < 0.01), the 2 vs 4 (0.5) ratio (mean ± SE = 9.67 ± 0.42; binomial test: *N* = 21, *P* < 0.01), and the 2 vs 5 (0.4) ratio (mean ± SE = 10.67 ± 0.50; binomial test: *N* = 21, *P* < 0.01). Performance on the SQD task decreased as the difference between the two quantities of food decreased (ie as the ratio increased) (Tables S1 to S3, Figure S1). Performance was not affected by any proxy of motivation (Table S3).

### Quantity discrimination playbacks

#### Vigilance

**Fig. 2.**
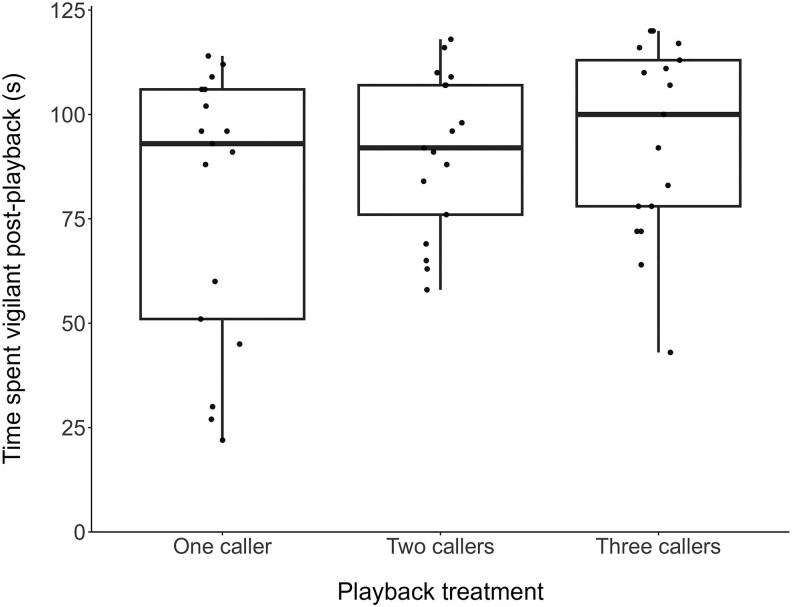
Time spent vigilant (seconds) in the 2 min following each playback treatment (*N* = 51 playbacks on 17 birds from 11 groups). Box plots show the median and 25th and 75th percentiles; whiskers indicate the values within 1.5 times the interquartile range. Points represent raw data.

**Fig. 3.**
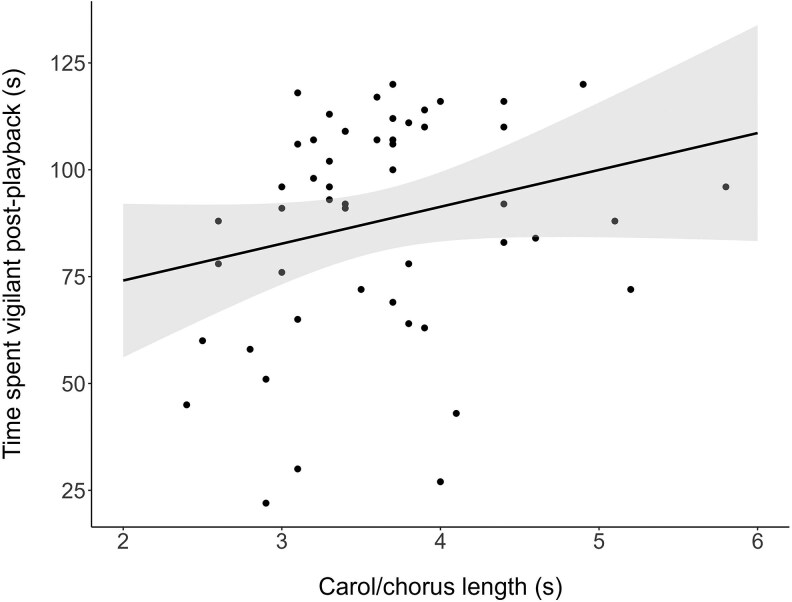
Time spent vigilant in the 2 min post-playback in relation to the length of carol or chorus presented (in seconds) (*N* = 51 playbacks on 17 birds from 11 groups). Points represent raw data; the shaded area represents 95% confidence intervals.

The time that individuals spent vigilant prior to the start of playbacks was not affected by any predictor tested (Table S4). The data revealed strong evidence that the time spent vigilant in the 2 min following playback was affected by playback treatment (Tables 1 and 2), with birds significantly more vigilant following playback of two or three callers compared to playback of one caller (Tables 1 and 2, Fig. 2). Post hoc analysis found no evidence that birds differed in the time spent vigilant following playback of two callers compared to three callers (Table 2). Magpies also spent more time vigilant following playback of a longer carol or chorus stimuli (Table 1, Fig. 3), though this effect was small (odds ratio = 1.11, 95% CI = 1.02–1.21).

**Table 1.** Top model set of candidate terms affecting the time spent vigilant in the 2 min post-playback. All models included group and bird ID as random terms. The top model set includes terms within 2 AICc of the best model. Coefficient estimates ± SE and 95% confidence intervals (CI) are given below the top model set. *N* = 51 trials on 17 birds from 11 groups. For the full model set of candidate terms tested, refer to Supplementary material (Table S5).

Top models	AICc	ΔAICc	
Playback treatment + Carol/chorus length	866.62	0.00	
*Basic*	963.94	97.32	

Parameter	Estimate	S. E	C.I
Carol/chorus length	0.10	0.04	0.02 to 0.19
Playback treatment			
One caller	0	…	…
Two callers	0.46	0.08	0.30 to 0.63
Three callers	0.60	0.09	0.43 to 0.78

**Table 2.** Post hoc analyses of the time spent vigilant in the 2 min following each playback track. Analysis was conducted on 51 playbacks on 17 magpies from 11 groups.

Contrast	Estimate	SE	*Z*-ratio	*P*	Odds ratio (C.I.)
One caller–two callers	−0.464	0.08	−5.52	<0.001	0.629 (0.517 to 0.766)
One caller–three callers	−0.602	0.09	−6.82	<0.001	0.548 (0.445 to 0.673)
Two callers–three callers	−0.139	0.08	−1.73	0.194	0.871 (0.721 to 1.050)

#### Songs and approach to speaker

**Fig. 4.**
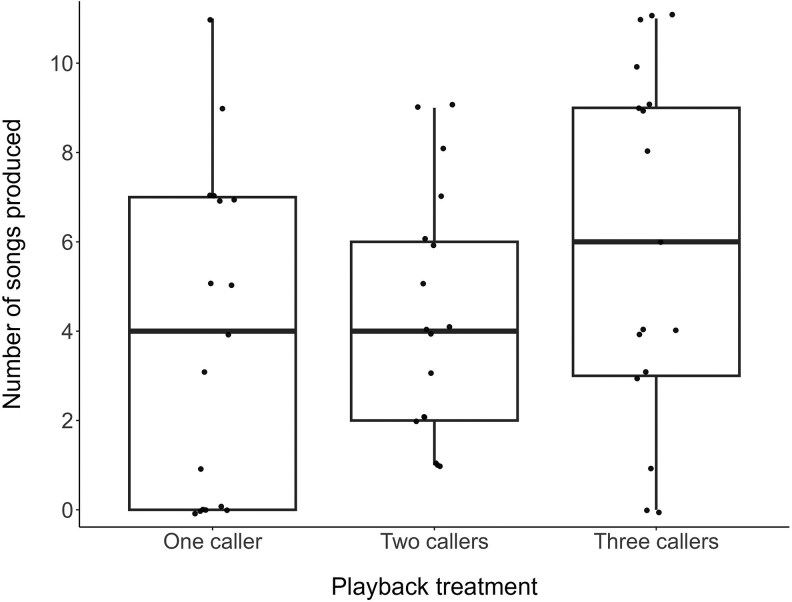
Number of songs sung in the 2 min following each playback treatment (*N* = 51 playbacks on 17 birds from 11 groups). Box plots show the median and 25th and 75th percentiles; whiskers indicate the values within 1.5 times the interquartile range. Points represent raw data.

Our findings reveal moderate evidence that individuals sung more songs in response to playback of three callers compared to playback of one caller (Table 3, Fig. 4), with a medium effect size (Table 4). Post hoc analysis revealed no evidence (*P* = 0.78) that birds sung more carols following playback of two callers compared to one caller, and only weak evidence (*P* = 0.08) that birds sung more calls following playback of three callers compared to two callers (Table 4). Carol/chorus length did not affect the number of songs sung in the 2 min post-playback (Table S6).

**Table 3.** Top model set of candidate terms affecting the number of songs sung in the 2 min post-playback. All models included group and bird ID as random terms. The top model set includes terms within 2 AICc of the best model. Coefficient estimates ± SE and 95% confidence intervals (CI) are given below the top model set. *N*  *=* 51 trials on 17 birds from 11 groups. For the full model set of candidate terms tested, refer to supplementary materials (Table S6).

Top models	AICc	ΔAICc	
Playback treatment	258.30	0.00	
*Basic*	263.90	5.60	

Parameter	Estimate	SE	CI
Playback treatment			
One caller	0	…	…
Two callers	0.11	0.17	−0.22 to 0.45
Three callers	0.45	0.16	0.14 to 0.75

**Table 4.** Post hoc analyses of the number of songs sung in the 2 min following each playback track. Analysis was conducted on 51 playbacks on 17 magpies from 11 groups.

Contrast	Estimate	SE	*z*-ratio	*P*	Rate ratio (CI)
One caller–two callers	−0.114	0.17	−0.68	0.778	0.892 (0.489 to 1.625)
One caller–three callers	−0.445	0.16	−2.82	0.013	0.641 (0.356 to 1.153)
Two callers–three callers	−0.331	0.15	−2.17	0.076	0.718 (0.718 to 0.401)

The likelihood of magpies approaching the speaker in the 2 min post-playback did not differ according to playback treatment, or any other predictor (Table S7).

### Linking SQD task performance and response to playbacks

Fifteen individuals presented with playbacks also completed cognitive testing, allowing for investigation of the relationship between performance in the cognitive task and response to playbacks.

**Fig. 5.**
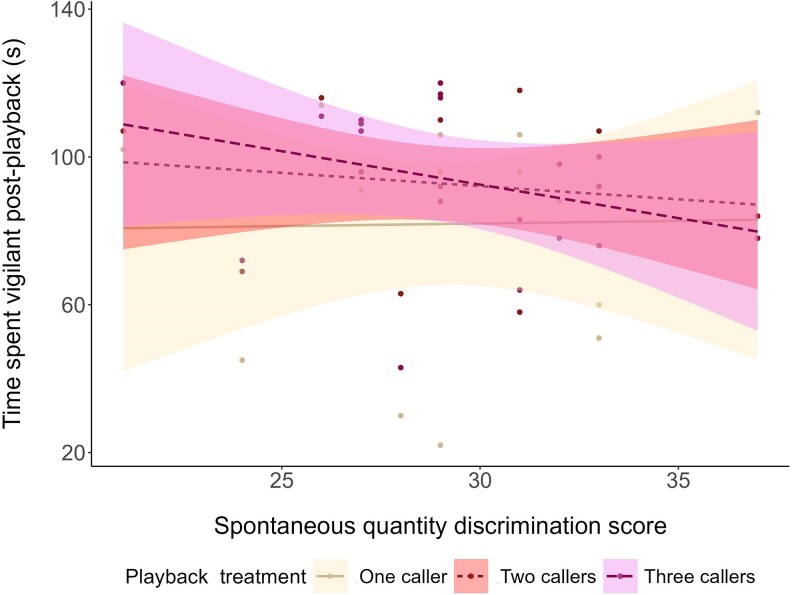
Time individuals spent vigilant in the 2-min post-playback in relation to the interaction between SQD score and playback treatment. Points represent raw data; the shaded areas represent 95% confidence intervals. *N* = 45 playbacks across 15 birds from 9 groups.

The time that individuals spent vigilant prior to the start of playbacks was not affected by SQD score, or any other predictor tested (Table S8). The time spent vigilant in the 2 min following playback was affected by the interaction between playback treatment and SQD score (Table 5, Fig. 5). Post hoc analysis revealed that following the three-caller playback track, individuals who performed better on the SQD task (chose the larger quantity of food more often) spent less time vigilant than individuals who performed worse on the task, and the size of this effect was moderate (Table 6, Fig. 5).

**Table 5.** Top model set of candidate terms affecting the time spent vigilant in the 2 min post-playback on a subset of individuals that had completed cognitive testing. All models included group and bird ID as random terms. The top model set includes terms within 2 AICc of the best model. Coefficient estimates ± SE and 95% confidence intervals (CI) are given below the top model set. *N*  *=* 45 trials on 15 birds from nine groups. For the full model set of candidate terms tested, refer to supplementary materials (Table S9).

Top models	AICc	ΔAICc	
Playback treatment * SQD score	772.48	0.00	
*Basic*	863.48	91.00	

Parameter	Estimate	SE	CI
Playback treatment			
One caller	0	…	…
Two callers	0.57	0.08	0.40 to 0.73
Three callers	0.70	0.08	0.53 to 0.87
SQD score	−0.04	0.24	−0.52 to 0.43
Playback treatment * SQD score			
One caller * SQD score	0	…	…
Two callers * SQD score	−0.25	0.09	−0.42 to −0.08
Three callers * SQD score	−0.53	0.09	−0.71 to −0.36

**Table 6.** Post hoc analyses of the time spent vigilant in the 2 min following each playback track on a subset of individuals that had completed cognitive testing. Analysis was conducted on 45 playbacks on 15 magpies from 9 groups.

Contrast	Estimate	SE	*z*-ratio	*P*	Odds ratio (CI)
One caller * SQD score	−0.04	0.24	−0.183	0.86	0.957 (0.597 to 1.534)
Two callers * SQD score	−0.30	0.24	−1.220	0.23	0.743 (0.461 to 1.197)
Three callers * SQD score	−0.58	0.24	−2.363	0.02	0.561 (0.348 to 0.906)

For individuals that had completed cognitive testing, there was no effect of SQD score on the number of songs focal birds produced (Table S10), or on the likelihood that individuals would approach the speaker following playback (Table S11).

## Discussion

In this study, we found that Western Australian magpies possess quantity discrimination abilities both when presented with a food-based SQD task, and when presented with playbacks of different numbers of extra-group magpie callers. We also found a relationship between performance on a SQD task and response to playback of three calling individuals. This represents the first evidence to date linking performance in a SQD task with behavioral response to playbacks of extra-group individuals.

### SQD performance and repeatability

When presented with the SQD task, magpies were able to discriminate between all ratios presented (2 vs 3, 2 vs 4, and 2 vs 5), and chose the larger food reward more often than would be expected at chance. These findings align with previous work on this study population (Hunter et al. 2025), and indicate that this species is capable of quantity discrimination, thus adding to the growing body of research documenting quantity discrimination abilities across taxa using cognitive tasks (Uller et al. 2003; Hunt et al. 2008; Uller and Lewis 2009; Jones and Brannon 2012; Bogale et al. 2014; Lucon-Xiccato et al. 2015; Bánszegi et al. 2016; Cox and Montrose 2016; Miletto Petrazzini et al. 2017; Szabo et al. 2021; Khatiwada and Burmeister 2022; Tomonaga et al. 2023).

The performance of magpies in the SQD task significantly improved as the difference between the two quantities of food increased (ie as the ratio decreased). The presence of this effect suggests magpies are employing the ANS to discriminate between quantities (Hyde 2011; Agrillo 2015). Similar increases in discrimination accuracy with decreasing ratio has been observed in several other species, including giraffes (*Giraffa camelopardalis*) (Caicoya et al. 2021), domestic rats (*Rattus norvegicus*) (Cox and Montrose 2016), chimpanzees (*Pan troglodytes*) (Tomonaga 2008), and prosimian primate species (Jones and Brannon 2012). While results from this study suggest magpies are employing the ANS to discriminate between quantities, further testing utilizing a greater range of quantity combinations (including both the same and different ratios to those presented here) is needed to confirm this.

We were able to calculate long-term repeatability for 18 adult magpies that had undergone testing on the SQD task at two different time points (2022 and 2023), and found that performance in this task was significantly repeatable (*R* = 0.461), suggesting our measures of SQD are robust, and not the result of confounding variables such as motivation, environmental factors, or energetic state (Ashton et al. 2022). While some previous studies have investigated long-term memory of learnt numerosity concepts (Burdyn et al. 1984; Beran 2004), the present study is the first, to our knowledge, to show that SQD abilities in a nonhuman animal may be repeatable in the long-term. Our finding of long-term repeatability of SQD performance provides evidence for the existence of a stable cognitive phenotype on which selection might act (Ashton et al. 2022).

It is important to note that our spontaneous quantity discrimination task did not eliminate the possibility that birds were using continuous cues (such as the volume or surface area of the food reward) rather than numerical cues when making their choice. The use of continuous cues is a potential confound in many spontaneous quantity discrimination tasks (Agrillo and Bisazza 2014; Khatiwada and Burmeister 2022; Simona et al. 2022; Tomonaga et al. 2023). To overcome this issue, future studies could present different numerical ratios with the same total surface area or volume, or could use quantity discrimination tasks involving training in addition to spontaneous choice tasks to gain a more robust understanding of an animal's numerical abilities (Agrillo and Bisazza 2014).

### Quantity discrimination playbacks

Our playback experiment revealed that magpies respond differently to songs sung by one caller, compared to two or three extra-group conspecifics. Magpies exhibited increased vigilance following the playback of two or three callers compared to playback of one caller and sung more in response to playback of three callers compared to playback of one. Whether magpies can distinguish between two and three callers is unclear. While there was no significant difference between these tracks in vigilance post-playback or in the number of songs sung post-playback (although post hoc analysis comparing the number of songs sung following the two-caller track and three-caller track was trending towards significance (*P* = 0.09)), the finding that magpies sing significantly more songs following playback of three but not two callers suggests that they are able to distinguish between two and three callers. The ability to assess the RHP of a rival group before entering into a contest with them is likely to be highly beneficial for social and territorial species (Parker 1974; McComb et al. 1994; Benson-Amram et al. 2017). In group-living species, the number of individuals in a group may be an important indicator of both threat level (as more individuals tend to represent a larger threat) and subsequently RHP (McComb et al. 1994; Radford 2003; Seddon and Tobias 2003; Benson-Amram et al. 2017). As such, it is likely that quantity discrimination abilities are under strong selective pressures in group-living species where the outcome of intergroup contests can be serious and potentially fatal (McComb et al. 1994; Radford 2003; Seddon and Tobias 2003). Western Australian magpies are a highly territorial species that often engages in intergroup disputes that can result in physical injury (Farabaugh et al. 1992; Hughes et al. 2003; Hidayat 2018), and thus the presence of quantity discrimination abilities in this species is not surprising. Our findings that magpies can differentiate between different numbers of extra-group callers aligns with previous research on territorial species such as lions (McComb et al. 1994), spotted hyenas (Benson-Amram et al. 2011), and subdesert mesites (Seddon and Tobias 2003).

The length of the extra-group song presented to focal birds had a weak effect on the time spent vigilant post-playback, with birds remaining vigilant for longer if presented with a longer carol or chorus. Song length has been related to territory defence, motivation, and mate quality in several bird species (Lambrechts and Dhondt 1987; Adhikerana and Slater 1993; Linhart et al. 2012; Ansell et al. 2020), with birds that sing longer songs generally considered to be more motivated or higher quality rivals (Lambrechts and Dhondt 1987; Adhikerana and Slater 1993; Ansell et al. 2020). Therefore, an increased vigilance response to these longer songs likely arises as magpies view longer songs as belonging to birds that represent a greater threat. It is important to note that we cannot rule out the possibility that magpies may be using other cues (such as song length, amplitude, or complexity) to discern the number of calling individuals in playback tracks, and hence the difference in response seen in our playbacks may be influenced by these vocal parameters. For example, the amplitude of three-caller choruses is generally louder (though not significantly) than one-caller carols, and magpies may use these differences in amplitude to help discern the number of intruders. However, these are cues that individuals would likely perceive and use in real intergroup interactions and hence are biologically relevant cues used for the discrimination of varying numbers of calling individuals. While the focus of our study was to investigate how magpies respond to real stimuli representing differing numbers of calling individuals, future studies could present songs of differing numbers of callers with the same length, number of notes, and amplitude to confirm that magpies are discerning between different numbers of callers, rather than differences in song parameters.

Interestingly, we found no evidence that the group size of the focal bird affected their response to playback. This contrasts with previous studies in lions and hyenas, where individuals were more likely to approach the speaker as the size of their own group increased (McComb et al. 1994; Benson-Amram et al. 2011). In the present study, group size ranged from three to 10 individuals (with an average of six individuals), meaning no focal bird belonged to a group smaller than the simulated intruder group, which may be why we did not see a difference in response based on group size. Playback simulation of intruding groups larger than the group size of the focal individual may elicit a stronger effect of group size on response, as larger focal groups may be more willing to approach these simulated larger groups compared to smaller focal groups.

### Linking SQD performance and playbacks

We found that performance in the SQD task was related to how magpies responded to playback; specifically, playback of three extra-group callers. Individuals that were more adept at choosing the larger quantity of food were less vigilant towards playback of three callers compared to individuals that performed worse. This contradicts our hypothesis, which predicted that individuals with a better SQD score would respond more strongly to three callers, which likely represent a greater threat. However, one potential explanation for the negative relationship between SQD score and playback response may be related to the unimodal nature of the signal presented (the playback). Multimodal signals may provide individuals with more information about a threat, or may increase the accuracy of information about that threat (Johnstone 1996; Partan and Marler 1999; Ronald et al. 2017). It is possible that birds that perform better on the SQD task (and who therefore also exhibit greater cognitive performance across other tasks; Blackburn et al. 2024) are more quickly able to integrate information from different sensory modalities, and discern that the playback chorus of three birds (an acoustic signal) is not associated with the presence of intruding conspecifics (a visual signal). These better performing birds may therefore not invest as much in vigilance and return to normal behaviors faster than birds that perform worse on the cognitive task, who maintain vigilance for longer as they try to assess the level of threat.

Alternatively, the difference in vigilance response between better and worse performing individuals on the SQD task could arise as the birds who exhibit increased vigilance following playback of three callers are birds that experience more inter-group interactions and conflict in their daily life and hence have heightened stress levels. Conflict is widely considered to be stressful for animals (von Holst 1998; Castles and Whiten 2010), and may result in heightened glucocorticoid levels (von Holst 1998; Wittig et al. 2015). As stress and glucocorticoids are known to affect cognitive performance (Mateo 2008; Reyes-Contreras and Taborsky 2022), individuals who experience higher rates of intergroup interactions and therefore respond to playbacks with heightened vigilance could also be experiencing decreased cognitive performance due to this heightened conflict and stress. Further investigation, including long-term monitoring of individuals to determine the rate of intergroup interactions, and measurement of fecal glucocorticoids to determine stress levels, would be helpful to disentangle the relationship between intergroup interactions, stress and cognitive performance in magpies.

Another alternative explanation for our findings is that the cognitive abilities utilized in the cognitive task and playback experiment may be underpinned by different cognitive domains. Performance on the SQD task presented in this study has previously been shown to be correlated with performance on other cognitive tasks within the physical domain (spatial memory and associative learning; Blackburn et al. 2024). Response to playback of extra group conspecifics may rely on cognitive abilities belonging to other cognitive domains, such as the processing of acoustic stimuli, or vocal discrimination (Wanker et al. 1998; Blackburn et al. 2022). Similarly, there may be a trade-off in this species between auditory processing (used in responding to vocalization and playbacks) and visual processing (used during the cognitive task), whereby individuals that perform better on the cognitive task are less adept at processing acoustic stimuli. Further research is needed to determine the reason behind the observed negative relationship between performance in these two tasks.

## Conclusion

In this study, we found evidence for quantity discrimination abilities in the territorial Western Australian magpie, both in a SQD task and a playback experiment. We also found that performance in a SQD task is related to the response of individuals to playback of three extra-group conspecific callers. Our findings not only add to the body of work documenting quantity discrimination abilities across species but also provide some of the first evidence that performance in SQD tasks can be related to ecologically relevant situations.

## Supplementary Material

araf093_Supplementary_Data

## Data Availability

Analyses reported in this article can be reproduced using the data provided by Blackburn et al. (2025).
