# Supplementary material for: Response to intruder number is related to spontaneous quantity discrimination performance in a wild bird
Source: Behav Ecol. 2025 Aug 20;36(5):araf093. doi: 10.1093/beheco/araf093 (PMC12449148; doi:10.1093/beheco/araf093)
Supplement: araf093_Supplementary_Data [file araf093_supplementary_data.docx]

**Response to intruder number is related to spontaneous quantity discrimination performance in a wild bird**

**SUPPLEMENTARY MATERIAL**

**SQD performance**

**Table S1.** Top model set of candidate terms affecting 2023 performance on spontaneous quantity discrimination task. All models included group and bird ID as random terms. The top model set includes terms within 2 AICc of the best model. Coefficient estimates ± S.E. and 95% confidence intervals (CI) are given below the top model set. *N =* 945 trials across 21 birds from 9 groups. For the full model set of candidate terms tested, refer to Supplementary Material (Table S2).

| **Top Models** | **AICc** | **ΔAICc** |  |
| --- | --- | --- | --- |
| Ratio | 1232.14 | 0 |  |
| *Basic model* | 1237.75 | 5.61 |  |
| **Parameter** | **Estimate** | **S. E** | **C.I** |
| Ratio  2 v 3 (0.67)  2 v 4 (0.5)  2 v 5 (0.4) | 0  0.29  0.52 | -  0.17  0.17 | -  -0.04, 0.61  0.19, 0.85 |

**Table S2.** Post-hoc analyses of 2023 performance on the spontaneous quantity discrimination task. *N =* 945 trials across 21 birds from 9 groups.

| **Contrast** | **Estimate** | **SE** | ***z-*ratio** | ***p*** | **Odds ratio (C.I)** |
| --- | --- | --- | --- | --- | --- |
| 2 v 3 – 2 v 4 | -0.286 | 0.16 | -1.73 | 0.194 | 0.751 (0.510, 1.107) |
| 2 v 3 – 2 v 5 | -0.520 | 0.17 | -3.08 | 0.006 | 0.594 (0.400, 0.883) |
| 2 v 4 – 2 v 5 | -0.234 | 0.17 | -1.37 | 0.359 | 0.791 (0.530, 1.182) |


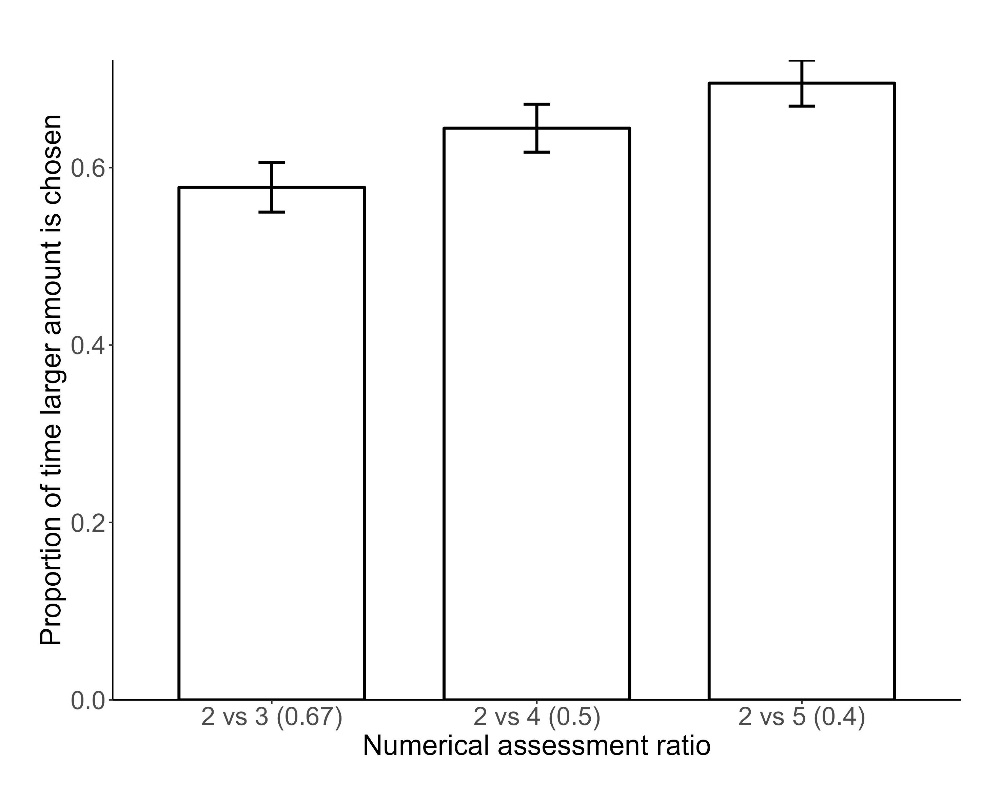


**Figure S1.** Mean (± SE) proportion of time the larger number of food items is chosen in the SQD task, at each ratio. *N* = 945 trials across 21 birds from 9 groups.

**Table S3.** Full model selection output for candidate terms affecting performance on the SQD task. All models included group and bird ID as random terms. Corrected Akaike information criterion (AICc) and ∆AICc are provided for each candidate model. Only within 2 AICc of the top model, and with predictors whose 95% confidence intervals did not intersect zero were included in the top model set and are highlighted in bold. *N =* 945 trials across 21 birds from 9 groups.

| **Models** | **AICc** | **∆AICc** |
| --- | --- | --- |
| **Ratio** | **1232.14** | **0.00** |
| Ratio * Adult group size^1^ | 1233.91 | 1.77 |
| Latency to interact with the task | 1235.88 | 3.74 |
| *Basic* | 1237.75 | 5.61 |
| Foraging effort | 1238.44 | 6.30 |
| Sex | 1239.19 | 7.05 |
| Temperature | 1239.24 | 7.10 |
| Weather | 1239.44 | 7.30 |
| Adult group size | 1239.74 | 7.60 |
| Body mass | 1239.76 | 7.62 |
| Foraging efficiency | 1239.77 | 7.63 |

^1^Although within 2AICc of the top model, not included in top model set as it is within 2AICc of a simpler model.

**Quantity discrimination playbacks**

**Table S4.** Full model selection output for candidate terms affecting time spent vigilant in the minute pre-playback. All models included group and bird ID as random terms. Corrected Akaike information criterion (AICc) and ∆AICc are provided for each candidate model. Only within 2 AICc of the top model, and with predictors whose 95% confidence intervals did not intersect zero were included in the top model set and are highlighted in bold. *N =* 51 playbacks on 17 birds from 11 groups.

| **Models** | **AICc** | **∆AICc** |
| --- | --- | --- |
| ***Basic*** | **480.58** | **0.00** |
| Track order^1^ | 482.37 | 1.79 |
| Sex^1^ | 482.48 | 1.90 |
| Adult group size | 482.84 | 2.26 |

^1^Although within 2AICc of the top model, not included in top model set as it is within 2AICc of the basic model.

**Table S5.** Full model selection output for candidate terms affecting time spent vigilant in the two minutes post-playback. All models included group and bird ID as random terms. Corrected Akaike information criterion (AICc) and ∆AICc are provided for each candidate model. Only within 2 AICc of the top model, and with predictors whose 95% confidence intervals did not intersect zero were included in the top model set and are highlighted in bold. *N =* 51 playbacks on 17 birds from 11 groups.

| **Models** | **AICc** | **∆AICc** |
| --- | --- | --- |
| **Playback treatment + Carol/chorus length** | **866.62** | **0** |
| Playback treatment | 870.24 | 3.62 |
| Playback treatment + Adult group size | 872.04 | 5.42 |
| Playback treatment * Adult group size | 875.01 | 8.39 |
| Carol/chorus length | 912.58 | 45.96 |
| Track order | 946.98 | 80.36 |
| Sex ratio | 955.00 | 88.38 |
| *Basic* | 963.94 | 97.32 |
| Adult group size | 965.53 | 98.91 |
| Sex | 965.72 | 99.10 |

**Table S6.** Full model selection output for candidate terms affecting the number of songs sung in the two minutes post-playback. All models included group and bird ID as random terms. Corrected Akaike information criterion (AICc) and ∆AICc are provided for each candidate model. Only within 2 AICc of the top model, and with predictors whose 95% confidence intervals did not intersect zero were included in the top model set and are highlighted in bold. *N =* 51 playbacks on 17 birds from 11 groups.

| **Models** | **AICc** | **∆AICc** |
| --- | --- | --- |
| **Playback treatment** | **258.30** | **0.00** |
| Sex | 263.51 | 5.21 |
| Playback treatment * Sex | 263.76 | 5.47 |
| *Basic* | 263.90 | 5.60 |
| Carol/chorus length | 263.93 | 5.63 |
| Playback treatment * Adult group size | 264.08 | 5.78 |
| Sex ratio | 264.98 | 6.69 |
| Adult group size | 265.90 | 7.60 |
| Track order | 266.46 | 8.17 |

**Table S7.** Full model selection output for candidate terms affecting the likelihood of individuals approaching the speaker in the two minutes post-playback. All models included group and bird ID as random terms. Corrected Akaike information criterion (AICc) and ∆AICc are provided for each candidate model. Only within 2 AICc of the top model, and with predictors whose 95% confidence intervals did not intersect zero were included in the top model set and are highlighted in bold. *N =* 51 playbacks on 17 birds from 11 groups.

| **Models** | **AICc** | **∆AICc** |
| --- | --- | --- |
| Carol/chorus length^1^ | 61.19 | 0.00 |
| ***Basic*** | **62.30** | **1.11** |
| Adult group size | 63.30 | 2.11 |
| Sex | 64.36 | 3.17 |
| Sex ratio | 64.55 | 3.36 |
| Playback treatment | 65.93 | 4.74 |
| Track order | 65.93 | 4.74 |
| Playback treatment * Adult group size | 72.06 | 10.87 |

^1^Although within 2AICc of the top model, not included in top model set as it is within 2AICc of the basic model and 95% confidence intervals intersect 0.

**Linking SQD task performance and response to playbacks**

**Table S8.** Full model selection output for candidate terms affecting time spent vigilant in the minute pre-playback on a subset of individuals that had completed cognitive testing. All models included group and bird ID as random terms. Corrected Akaike information criterion (AICc) and ∆AICc are provided for each candidate model. Only within 2 AICc of the top model, and with predictors whose 95% confidence intervals did not intersect zero were included in the top model set and are highlighted in bold. *N* = 45 playbacks on 15 birds from 9 groups.

| **Models** | **AICc** | **∆AICc** |
| --- | --- | --- |
| ***Basic*** | **414.40** | **0.00** |
| SQD score^1^ | 415.55 | 1.15 |
| Track order^1^ | 416.23 | 1.83 |
| Adult group size | 416.58 | 2.18 |
| Sex | 416.79 | 2.39 |

^1^Although within 2AICc of the top model, not included in top model set as it is within 2AICc of the basic model.

**Table S9.** Full model selection output for candidate terms affecting time spent vigilant in the two minutes post-playback on a subset of individuals that had completed cognitive testing. All models included group and bird ID as random terms. Corrected Akaike information criterion (AICc) and ∆AICc are provided for each candidate model. Only within 2 AICc of the top model, and with predictors whose 95% confidence intervals did not intersect zero were included in the top model set and are highlighted in bold. *N* = 45 playbacks on 15 birds from 9 groups.

| **Models** | **AICc** | **∆AICc** |
| --- | --- | --- |
| **Playback treatment * SQD** **score** | **772.48** | **0.00** |
| Playback treatment + Carol/chorus length | 786.17 | 13.69 |
| Carol/chorus length | 795.09 | 22.61 |
| Playback treatment * Adult group size | 798.59 | 26.11 |
| Playback treatment | 802.00 | 29.51 |
| Playback treatment + SQD score | 803.38 | 30.90 |
| Playback treatment + Adult group size | 803.92 | 31.44 |
| Track order | 851.94 | 79.46 |
| Sex ratio | 859.88 | 87.40 |
| *Basic* | 863.48 | 91.00 |
| Sex | 864.57 | 92.09 |
| SQD score | 864.59 | 92.11 |
| Adult group size | 865.15 | 92.67 |

**Table S10.** Full model selection output for candidate terms affecting the number of songs sung in the two minutes post-playback on a subset of individuals that had completed cognitive testing. All models included group and bird ID as random terms. Corrected Akaike information criterion (AICc) and ∆AICc are provided for each candidate model. Only within 2 AICc of the top model, and with predictors whose 95% confidence intervals did not intersect zero were included in the top model set and are highlighted in bold. *N* = 45 playbacks on 15 birds from 9 groups.

| **Models** | **AICc** | **∆AICc** |
| --- | --- | --- |
| **Playback treatment** | **225.27** | **0** |
| Carol/chorus length | 228.89 | 3.62 |
| Sex | 229.02 | 3.75 |
| Sex ratio | 229.64 | 4.37 |
| *Basic* | 229.65 | 4.38 |
| Track order | 230.10 | 4.83 |
| Adult group size | 231.84 | 6.57 |
| SQD score | 231.85 | 6.58 |
| Playback treatment * Adult group size | 233.29 | 8.02 |
| Playback treatment * SQD score | 234.59 | 9.32 |

**Table S11.** Full model selection output for candidate terms affecting the likelihood of individuals approaching the speaker in the two minutes post-playback on a subset of individuals that had completed cognitive testing. All models included group and bird ID as random terms. Corrected Akaike information criterion (AICc) and ∆AICc are provided for each candidate model. Only within 2 AICc of the top model, and with predictors whose 95% confidence intervals did not intersect zero were included in the top model set and are highlighted in bold. *N* = 45 playbacks on 15 birds from 9 groups.

| **Models** | **AICc** | **∆AICc** |
| --- | --- | --- |
| Carol/chorus length^1^ | 54.64 | 0.00 |
| ***Basic*** | **56.05** | **1.41** |
| Adult group size | 57.36 | 2.72 |
| Sex | 57.88 | 3.25 |
| Sex ratio | 58.10 | 3.46 |
| SQD score | 58.36 | 3.73 |
| Playback treatment | 59.76 | 5.12 |
| Track order | 59.76 | 5.12 |
| Playback treatment * Adult group size | 65.97 | 11.33 |
| Playback treatment * SQD score | 67.21 | 12.58 |

^1^Although within 2AICc of the top model, not included in top model set as it is within 2AICc of the basic model, and confidence intervals intersect 0.
